# *Lactobacillus* probiotics potential in *Blastocystis infection*: in vitro and in vivo studies

**DOI:** 10.1186/s13099-025-00765-6

**Published:** 2025-12-13

**Authors:** Mona Gamal Baz Mohamed, Ibrahim A. Aboul Asaad, Dareen Abd Elaziz Mohamed Ali, Dalia Abdelmageed Ahmad Elmehy, Sarah M. Abdo

**Affiliations:** 1 Medical Parasitology department, Faculty of Medicine, Kafrelsheikh University, Kafr El-Sheikh, Egypt; 2 Medical Parasitology, Faculty of Medicine, Tanta University, Tanta, Egypt; 3 Pathology Department, Faculty of Medicine, Tanta University, Tanta, Egypt

**Keywords:** Blastocystis, Metronidazole, Probiotics, Histopathology, IgA expression

## Abstract

*Blastocystis* is a widespread intestinal parasite with debated pathogenicity. *Blastocystis* infection often persists despite metronidazole therapy, highlighting the need for adjunctive strategies. This study evaluated the therapeutic efficacy of metronidazole, *Lactobacillus* probiotics, and their combination against *Blastocystis* infection using in vitro and in vivo models. In vitro cultures of *Blastocystis* were treated with metronidazole (10 µg/mL), probiotics (10⁸ CFU/mL), or both. Viability and parasite counts were assessed at 24 and 48-hours post-treatment. In vivo, infected mice received metronidazole (20 mg/kg), probiotics (10⁹ CFU/day), or both for 7 days. Parasitological, histopathological, immunohistochemical, and cytokine evaluations were conducted. At 48 h in vitro, metronidazole reduced *Blastocystis* count by 88.6% and viability by 91.3%; probiotics reduced count by 87.2% and viability by 90.6%. The combination achieved 94.8% and 96.8% reductions, respectively (*p* < 0.001). In vivo, stool cysts decreased by 86% (metronidazole), 84% (probiotics), and 98.5% (combined). Intestinal cysts decreased by 85.1%, 82.9%, and 98.5%, respectively. Histological improvements and restoration of IgA-secreting cells were most prominent in the combined group. Pro-inflammatory cytokines (IL-1β, IL-6, IFN-γ) decreased most with combination therapy—by 66.9%, 57.8%, and 60.1%, respectively—compared to untreated controls (*p* < 0.001). These findings indicate that probiotics enhance the efficacy of metronidazole, supporting their role as a promising adjunctive therapy for *Blastocystis* infection. The combined treatment yielded the most profound parasitological, immunological, and histological improvements, supporting its potential as a superior therapeutic strategy.

## Introduction

*Blastocystis* is a widespread intestinal protozoan that infects a significant proportion of the global population, with prevalence rates ranging from 2% to over 50% depending on geographic location and diagnostic methods [1]. Despite its high prevalence, the role of *Blastocystis* in human health remains controversial. While some studies associate *Blastocystis* with gastrointestinal disorders such as irritable bowel syndrome (IBS) and diarrhea, others suggest that it may be a harmless commensal organism within the human gut microbiome [2].

In addition to its high prevalence in humans, *Blastocystis* is also considered a parasite of zoonotic potential. It has been identified in a broad range of non-human hosts, including livestock (cattle, pigs, sheep, goats, and camels), companion animals (dogs and cats), rodents, and birds [1]. Higher infection rates have been reported among individuals with close contact with animals, such as farmers and slaughterhouse workers, suggesting occupational risk [1, 3].

Current treatment options for *Blastocystis* infection typically involve antiparasitic drugs like MTZ and nitazoxanide. However, these treatments are often hindered by inconsistent efficacy, side effects, and the potential for drug resistance [4]. The challenges associated with conventional therapies have prompted research into alternative treatment modalities, including the use of probiotics - live microorganisms that provide health benefits when consumed in adequate amounts [5].

Probiotics have gained attention for their ability to modulate the gut microbiota, enhance mucosal barrier function, and interact with the host immune system [6]. *Lactobacilli* have been shown to transiently colonize the human colon, with strains such as *L. rhamnosus* GG adhering to colonic mucosa following oral administration. Evidence also indicates that common dairy-derived species, including *L. delbrueckii*, can modulate inflammatory pathways and restore mucosal balance in experimental colitis models, highlighting their potential therapeutic role in intestinal inflammation [7, 8]. These properties have led to investigations into their potential use against various gastrointestinal pathogens, including protozoan parasites like *Giardia lamblia* and *Entamoeba* histolytica [9, 10]. *Lactobacillus* includes numerous strains however, *Lactobacillus fermentum* generate substantially higher levels of hydrogen peroxide (H₂O₂) enhancing direct oxidative damage to the parasites [11]. Although the research on probiotics specifically targeting *Blastocystis* is still in its early stages, preliminary studies suggest that certain probiotic strains may inhibit the growth of *Blastocystis* and ameliorate associated symptoms [12].

This study aimed to evaluate and compare the therapeutic efficacy of MTZ, *Lactobacillus delbrueckii* and *Lactobacillus fermentum* probiotics, and their combination with MTZ against *Blastocystis* infection using both in vitro and in vivo models, with a focus on parasitological, histopathological, immunohistochemical, and immunological outcomes.

## Materials and methods

### Study design and ethical approval

This study was conducted in the Medical Parasitology Department, Faculty of Medicine, Tanta University, from August 2023 to August 2024. Ethical approval was obtained for all experimental procedures, including the use of animals, from the Research Ethics Committee of the Faculty of Medicine, Tanta University (Approval Code: 36265/8/23). All animal handling and interventions were performed according to the institutional guidelines for animal care and the National Institutes of Health (NIH) Guide for the Care and Use of Laboratory Animals.

### Parasite isolation and culture

*Blastocystis* was isolated from a fresh stool sample collected from a patient suffering from irritable bowel syndrome (IBS) at the Tropical Medicine Department, Tanta University Hospital. The sample was processed following the protocols of Irikov et al. (2009) [13]. Briefly, stool was emulsified in saline, filtered, centrifuged at 1000 rpm for 2 min, and inoculated into Jones’ medium (1 ml sediment + 3 ml medium). *Blastocystis* counts were determined at 24 and 48 h post-treatment using a Neubauer hemocytometer under ×40 light microscopy, with five replicates per sample averaged. Parasite viability was assessed by Trypan Blue exclusion, where intact cells remained unstained and non-viable cells appeared blue, and the percentage of viable parasites was calculated accordingly [13, 14].

$$\:\text{Viability}\:{(\%)}=\frac{\text{Number of viable parasites}}{\text{Total parasites}}\times\:100$$

### Drug and probiotic preparation

Metronidazole (MTZ) was administered as Flagyl^®^ suspension (125 mg/5 mL; SANOFI-Pharmaceuticals, Cairo, Egypt) [15]. *Lactobacillus* probiotics for both in vivo and in vitro experiments were sourced from Lacteol Fort^®^ (Rameda, Egypt), containing *Lactobacillus delbrueckii* and *Lactobacillus fermentum* [16]. Each sachet contained 10 billion (10¹⁰) lyophilized organisms with excipients. For in vivo administration, the contents of one sachet were dissolved in 1 mL of sterile distilled water, and 0.1 mL (equivalent to 1 × 10⁹ CFU) was administered orally to mice daily for seven consecutive days [17]. For in vitro testing, the sachet contents were reconstituted in sterile distilled water to achieve a final concentration of 1 × 10⁹ CFU/mL [18, 19].

### In vitro evaluation

Cultures containing approximately 1 × 10^3^ to 1 × 10^5^ *Blastocystis* organisms/mL were used for drug susceptibility testing [13, 14]. Four experimental groups were prepared in vitro: Culture I (Control): Untreated *Blastocystis*, Culture II (MTZ-treated): MTZ at 120 µg/mL [18], Culture III (Probiotic-treated): *Lactobacillus* at 1 × 10⁹ CFU/mL [19], Culture IV (Combined): MTZ + Lactobacillus [20, 21]. Each group included four replicate tubes, incubated at 37 °C. Parasite viability was assessed at 24 and 48-hours post-treatment using 0.4% Trypan blue stain. Fifteen microliters of culture fluid were mixed with equal volume stain and observed under light microscopy after 15 min [18, 22]. Growth inhibition percentage was calculated based on viable parasite counts. Ultrastructural Analysis: One mL of each culture was fixed in 4% glutaraldehyde with 0.1 M sodium cacodylate buffer and processed for scanning and transmission electron microscopy (SEM and TEM) [23].

### Animals and infection

Fifty male Swiss albino mice (10 weeks old, 25–30 g) were acclimatized for 4 days under standard laboratory conditions with ad libitum access to food and water. Animals were housed according to institutional animal care standards [24]. All mice were housed individually in separate sterilized cages to prevent cross-contamination between experimental groups. Cages were cleaned and disinfected regularly, and animals were provided with sterilized bedding, food, and water ad libitum. As experimental susceptibility to *Blastocystis* is strain dependent, and swiss albino mice are considered permissive hosts that reliably sustain infection [22]. Each mouse was orally infected with 0.25 mL suspension containing 10,000 *Blastocystis* cysts via intraesophageal gavage [25]. Infection was confirmed via stool examination 3 days post-infection.

### Treatment groups

Three weeks post-infection, mice were randomly divided into five groups (*n* = 10 each): Group I: Non-infected, non-treated control, Group II: Infected, non-treated, Group III: Infected, treated with MTZ (120 µg/kg/day) [18, 21], Group IV: Infected, treated with Lactobacillus (1 billion CFU/day) [26]. Group V: Infected, treated with combined MTZ and Lactobacillus [27]. Treatments were administered via intraesophageal gavage for seven consecutive days.

### Post-treatment evaluations

On day 8 post-treatment, mice were anesthetized with thiopental sodium (1 g/20 mL; 0.5 mL/mouse) and sacrificed for the following assessments.

- **Parasitological assessment**: Stool samples were collected and centrifuged at 1000 rpm for 2 min, and sediments were examined microscopically to quantify *Blastocystis* cysts [22]. Intestines were excised, washed, and sectioned. Some segments were fixed in 10% formalin for histology. The rest were soaked in 37 °C saline for 30 min, centrifuged at 1500 rpm for 2 min, and parasite counts were performed under 10× magnification in five high-power fields [28].
- **Histopathological and immunohistochemical evaluation**: Formalin-fixed intestinal tissues were paraffin-embedded, sectioned at 5 μm, and stained with hematoxylin and eosin (H&E) for histological assessment [18]. For immunohistochemistry, sections were stained with fluorescein isothiocyanate (FITC)-conjugated anti-mouse IgA antibodies (1:100; Sigma-Aldrich) to detect IgA-secreting cells in small and large intestines [29].
- **Biochemical assessment** : Serum levels of interleukin-1 beta (IL-1β), interleukin-6 (IL-6), and interferon-gamma (IFN-γ) were quantified using enzyme-linked immunosorbent assay (ELISA) kits (Sun Red Biotechnology, Shanghai), following the manufacturer’s protocols [30]. Blood samples were collected from all mice via cardiac puncture at the end of the experiment, and sera were separated by centrifugation at 3000 rpm for 15 min and stored at − 80 °C until analysis. ELISA procedures were performed according to the manufacturer’s instructions, and absorbance was measured using a microplate reader at the appropriate wavelength.
- **Statistical analysis**: Data were analyzed using Statistical Program for Social Science (SPSS) version 22.0 Quantitative data were expressed as mean ± standard deviation (SD). Mean value, Standard Deviation [SD], F-test was applied to compare the significant differences between the studied groups, Growth inhibition (%) and Analysis of variance [ANOVA] tests (f).

## Results

### In vitro efficacy of treatments against *Blastocystis*

After 24 h of drug cessation, all treatments significantly reduced *Blastocystis* count and viability compared to the non-treated group (*p* < 0.001). The non-treated culture showed a count and viability of 102.0 ± 8.18. MTZ reduced the count by 76.5% and viability by 81.6%; probiotics reduced count by 73.5% and viability by 79.4%; the combination therapy achieved the highest reduction (85.7% in count and 88.0% in viability). The combination significantly outperformed either treatment alone, though no significant difference was observed between MTZ and probiotics individually (*p* > 0.05).

**Fig. 1:**
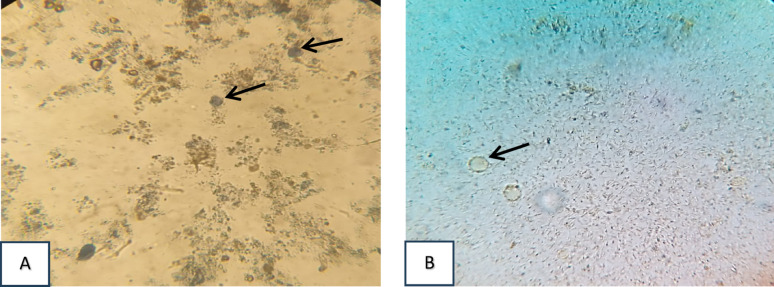
(**A & B**) shows representative light microscopy images of *Blastocystis* stained with trypan blue dye. Non-viable cells appear stained in blue (Fig. 1A), whereas viable cells remain unstained and appear bright (Fig. 1B). All observed *Blastocystis* cells are non-viable (**A**). All observed *Blastocystis* cells are viable (**B**) (X40 magnification)

At 48 h post-treatment, the initial *Blastocystis* count and viability were 112.40 ± 8.50 in the non-treated group. All treatments significantly reduced both parameters (*p* < 0.001). MTZ reduced count by 88.6% and viability by 91.3%, while probiotics reduced count by 87.2% and viability by 90.6%. The combination treatment was most effective, reducing count by 94.8% and viability by 96.8%, significantly outperforming individual treatments. However, no significant difference was observed between MTZ and probiotics alone (*p* > 0.05). (Table 1).

**Table 1:** ***Blastocystis*** count and viability after 24 and 48 h of cessation of drug administration

		Range	Mean ± SD	% of reduction	F. test	*p*. value	Post Hock test			
* Baseline (before treatment)	all cultures	95–120	108.5 ± 7.6				-	-	-	-
Total count after 24hs	Culture I	91–113	102.0 ± 8.18		281.749	0.001*	P1	0.001*	P4	0.456
	Culture II	21–30	24.40 ± 3.51	76.5%			P2	0.001*	P5	0.002*
	Culture III	21–32	27.0 ± 4.85	73.5%			P3	0.001*	P6	0.011*
	Culture IV	11–20	14.60 ± 3.36	85.7%						
Viability after 24 hs	Culture I	91–113	102.0 ± 8.18		413.294	0.001*	P1	0.001*	P4	0.684
	Culture II	15–21	18.8 ± 2.28	81.6%			P2	0.001*	P5	0.009*
	Culture III	16–24	21.0 ± 3.16	79.4%			P3	0.001*	P6	0.040*
	Culture IV	10–15	12.2 ± 1.92	88.0%						
Total count after 48hs	Culture I	98–120	112.40 ± 8.50		634.218	0.001*	P1	0.001*	P4	0.853
	Culture II	11–15	12.80 ± 1.48	88.6%			P2	0.001*	P5	0.008*
	Culture III	12–18	14.40 ± 2.30	87.2%			P3	0.001*	P6	0.026*
	Culture IV	4–7	5.80 ± 1.30	94.8%						
Viability after 48hs	Culture I	98–120	112.40 ± 8.50		718.693	0.001*	P1	0.001*	P4	0.776
	Culture II	9–11	9.80 ± 0.84	91.3%			P2	0.001*	P5	0.022*
	Culture III	9–13	10.6 ± 1.52	90.6%			P3	0.001*	P6	0.039*
	Culture IV	3–5	3.60 ± 0.89	96.8%						

*Baseline count represents the mean number of *Blastocystis* cells in untreated cultures prior to drug administration, serving as the reference for calculating percentage reduction.

P1: Culture I & Culture II

P2: Culture I & Culture III

P3: Culture I & Culture IV

P4: Culture II & Culture III

P5: Culture II & Culture IV

P6: Culture III & Culture IV

### Ultrastructural changes observed by SEM

Figure 3 shows SEM at 24 h, showed *Blastocystis* cysts in the control group (Culture I) with smooth surfaces with fibrous coats. MTZ-treated cysts (Culture II) had patchy surface disruptions, while probiotic-treated ones (Culture III) exhibited extensive folding. The combined treatment (Culture IV) caused increased folding, blebbing, and surface irregularities.

**Fig. 2:**
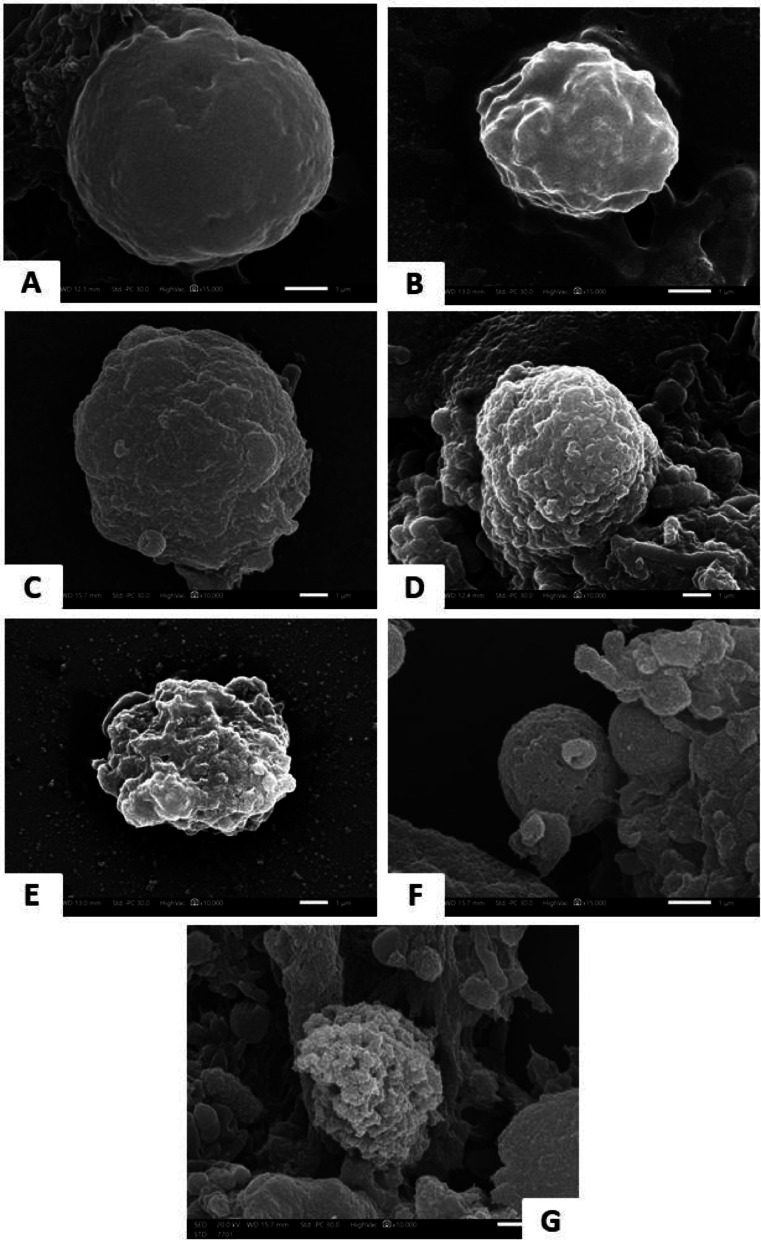
Scanning electron micrographs of *Blastocystis* after 24 hours under different treatments (**A&B**) Untreated control showing oval shape and smooth surface (×15,000). (**C&D**) MTZ-treated culture exhibiting spherical forms with patchy, broken surfaces and surface folding (×10,000). (**E**) Probiotic-treated culture with marked surface convolution (×10,000). (**F **(×15,000)& G (×10,000)) Combination treatment showing extensive folding, blebbing, and disrupted surface morphology.

At 48 h, Culture II showed deep furrows and blebbing; Culture III showed membrane pores; Culture IV displayed severe disruption, including compromised membrane integrity and damaged cytoplasmic content, indicating synergistic damage from combined therapy. These SEM images are representative observations; however, the proportion of cysts exhibiting each morphology was not quantified, and further studies are required to determine the prevalence of these structural changes. (Fig. 2)

### Ultrastructural changes observed by TEM

**Fig. 3:**
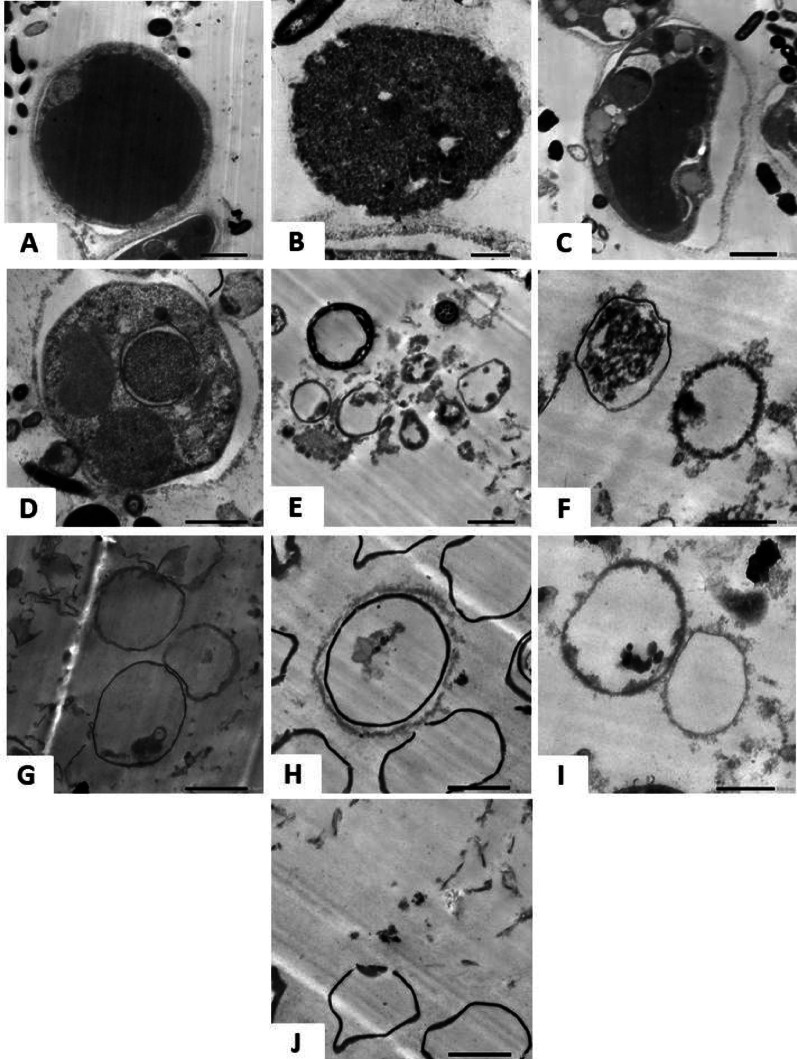
TEM images illustrate morphological variations of *Blastocystis* under different treatments. At 24 hours, untreated organisms appear in vacuolar (**A**), granular (**B**), amoeboid (**C**), and multi-vacuolar forms (**D**). In MTZ-treated culture (**E**), the vacuolar form dominates with mild electron-dense particles. Lactobacillus-treated culture (**F**) shows a dominant amoeboid form with similar particle presence. Combined MTZ and probiotic treatment (**G**) reveals mostly vacuolar forms with a clear central vacuole. After 48 hours, MTZ-treated (**H**) and probiotic-treated (**I**) cultures both exhibit vacuolar forms with few electron-dense particles. The combined treatment (**J**) also shows vacuolar forms with membrane rupture and absence of electron-dense particles. Magnification: X3000

Figure 3 shows vacuolar *Blastocystis* in Culture I (round/oval with a central vacuole and thin cytoplasm; Fig. 3A), granular (central granule-filled body; Fig. 3B), amoeboid (irregular shape; Fig. 3C), and multivacuolar forms (Fig. 3D). After 24 h, Culture II showed a predominance of the vacuolar form with mild electron-dense particles (Fig. 3E). Culture III showed mostly amoeboid forms with mild electron-dense material (Fig. 3F). Culture IV exhibited primarily vacuolar forms with disrupted plasma membranes and loss of intracellular content (Fig. 3G). At 48 h, Culture II retained vacuolar forms with minimal electron-dense particles (Fig. 3H), Culture III presented amoeboid forms with electron-dense granules (Fig. 3I), and Culture IV showed vacuolar forms with marked membrane rupture and cytoplasmic leakage (Fig. 3J), indicating progressive structural damage.

### In Vivo stool and intestinal counts of *Blastocystis*

Regarding *Blastocystis* count in mice stool, as shown in Table 2, Group II had a high stool count of *Blastocystis* (148,540 ± 25,830.37 cysts/g). Group V showed the greatest reduction (98.5%), while Groups III and IV had reductions of 86% and 84%, respectively. The combined treatment was significantly more effective than either agent alone (*p* < 0.001), with no significant difference between Groups III and IV (*p* > 0.05).

In the intestinal wash, Group II had a mean count of 7.78 ± 1.21 cysts/HPF. Groups III and IV showed reductions of 85.1% and 82.9%, respectively. Group V achieved complete elimination (98.5% reduction), with statistically significant differences compared to Groups II, III, and IV (*p* < 0.001), while no significant difference was found between Groups III and IV.

**Table 2:** Mean count of *Blastocystis* per gram (1 gm) in mice stool and in the intestinal wash/HPF across infected control and treated groups

		Range	Mean ± SD	% of reduction	F. test	*p*. value	Post Hock test			
Mice stool	Group II	125,700–190,000	148,540 ± 25830.37		121.475	0.001*	P1	0.001*	P4	0.749
	Group III	17,000–26,000	20,800 ± 3962.32	86.0%			P2	0.001*	P5	0.025*
	Group IV	19,000–37,000	23,600 ± 7536.57	84.1%			P3	0.001*	P6	0.048*
	Group V	900–4900	2310 ± 1656.96	98.4%						
Intestinal wash/HPF	Group II	6.2–9.1	7.78 ± 1.21		34.324	0.001*	P1	0.001*	P4	0.682
	Group III	0.78–1.4	1.16 ± 0.24	85.1%			P2	0.001*	P5	0.008*
	Group IV	0.96–1.5	1.33 ± 0.22	82.9%			P3	0.001*	P6	0.019*
	Group V	0.01–0.2	0.12 ± 0.08	98.5%						

P1: G II & G III

P2: G II & G IV

P3: G II & G V

P4: G III & G IV

P5: G III & G V

P6: G IV & G V

### Histopathological findings

**Fig. 4:**
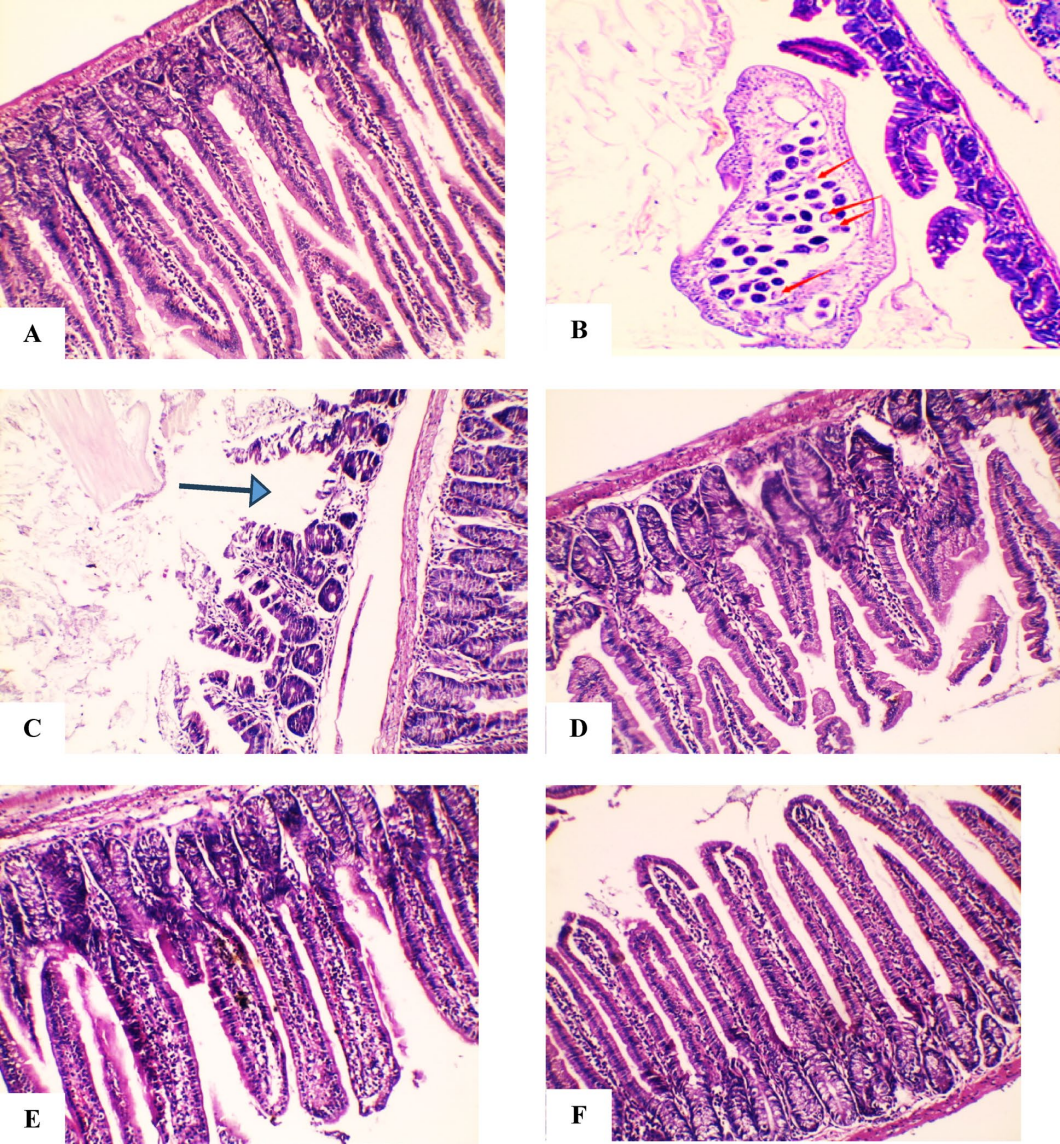
Histological changes in the small intestine under light microscopy. The healthy control (**A**) has normal mucosa. The infected, untreated group (**B, C**) displays *Blastocystis *cysts (red arrows), mucosal ulcers, and villous atrophy. MTZ-treated (**D**) and probiotics-treated (**E**) groups show improved villous structure, while the combined treatment group (**F**) exhibits nearly normal villi. (*All stained with H&E; magnifications: 200x & 400x.*)

Small Intestine: Group I displayed normal villous architecture with no inflammation (Fig. 4A). Group II showed mucosal ulceration, villous atrophy, and luminal Blastocystis cysts (Fig. 4B–C). Group III (MTZ) had moderate mucosal healing and partial preservation of villi (Fig. 4D). Group IV (probiotics) showed moderate restoration of mucosa and villous structure (Fig. 4E). Group V (MTZ + probiotics) exhibited complete mucosal healing and enhanced villous architecture (Fig. 4F).

**Fig. 5:**
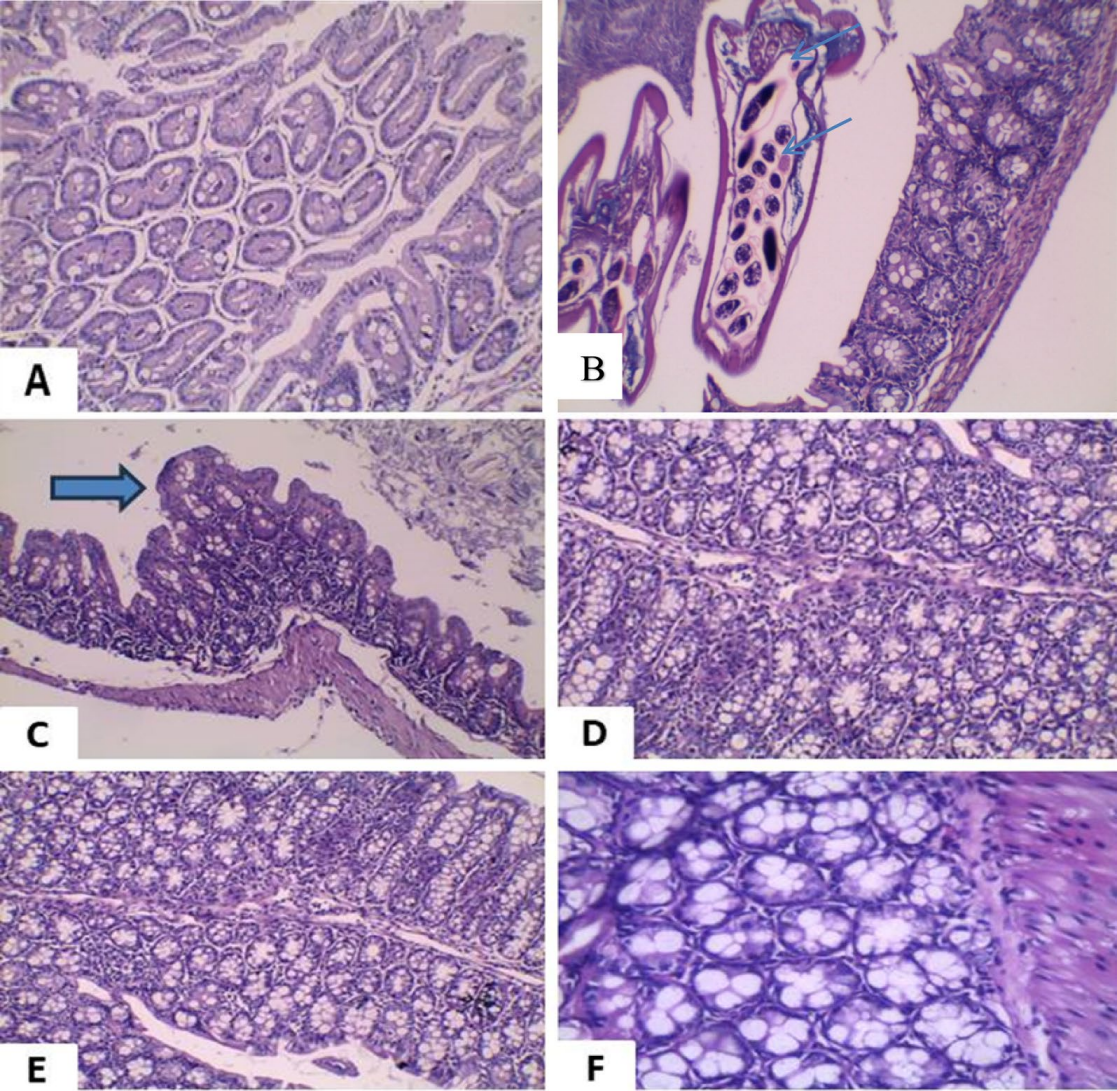
Histological evaluation of the large intestine under light microscopy. The control (**A**) appears normal. Infected, untreated tissues (**B, C**) show *Blastocystis* cysts (blue arrows) and crypt hyperplasia. MTZ (**D**) and probiotics (**E**) reduce inflammation and eliminate cysts. The combined therapy group (**F**) reveals mucosal architecture. (*All stained with H&E; magnifications: 200x & 400x.*)

Large Intestine: Group I revealed normal mucosa (Fig. 5A), while Group II showed crypt distortion, dense inflammatory infiltration, and Blastocystis cysts (blue arrow) (Fig. 5B–C). Group III exhibited near-normal crypts with moderate inflammation (Fig. 5D). Group IV showed mucosal restoration and mild to moderate inflammation (Fig. 5E). Group V demonstrated complete crypt healing with preserved architecture and only scattered inflammatory cells (Fig. 5F).

### Immunohistochemical detection of IgA-Secreting cells

**Fig. 6:**
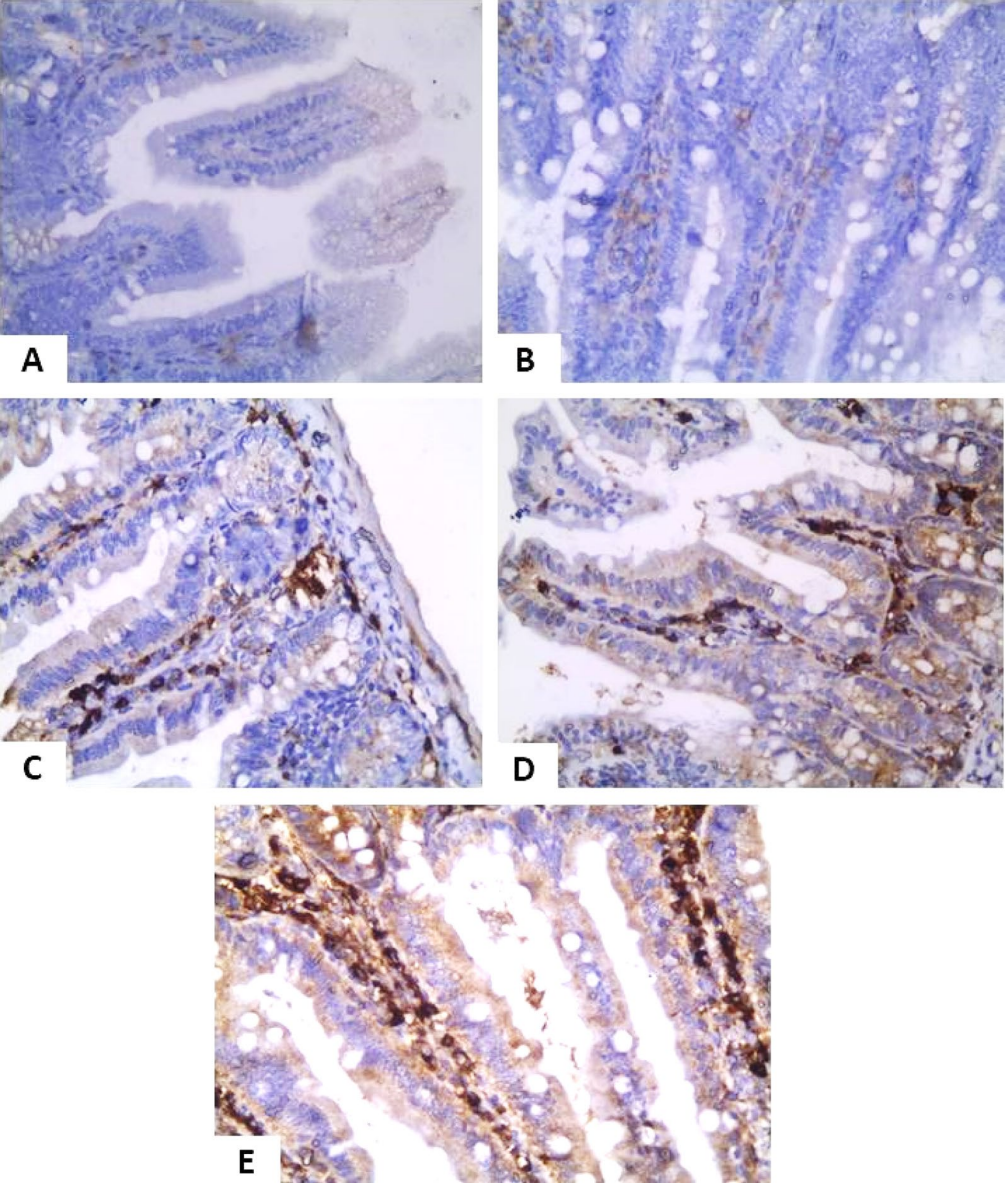
Immunohistochemical staining of the small intestine for IgA expression. Both the non-infected control (**A**) and infected, non-treated group (**B**) lack IgA secretory cells. MTZ-treated (**C**) and probiotics-treated (**D**) groups show mild to moderate increases in IgA expression, along with nonspecific staining of inflammatory infiltrates. The combined treatment group (E) exhibits a marked rise in IgA-secreting cells and prominent inflammatory infiltration. (All images at 400x magnification; H&E stain with nonspecific IgA staining in inflammatory cells

**Fig. 7:**
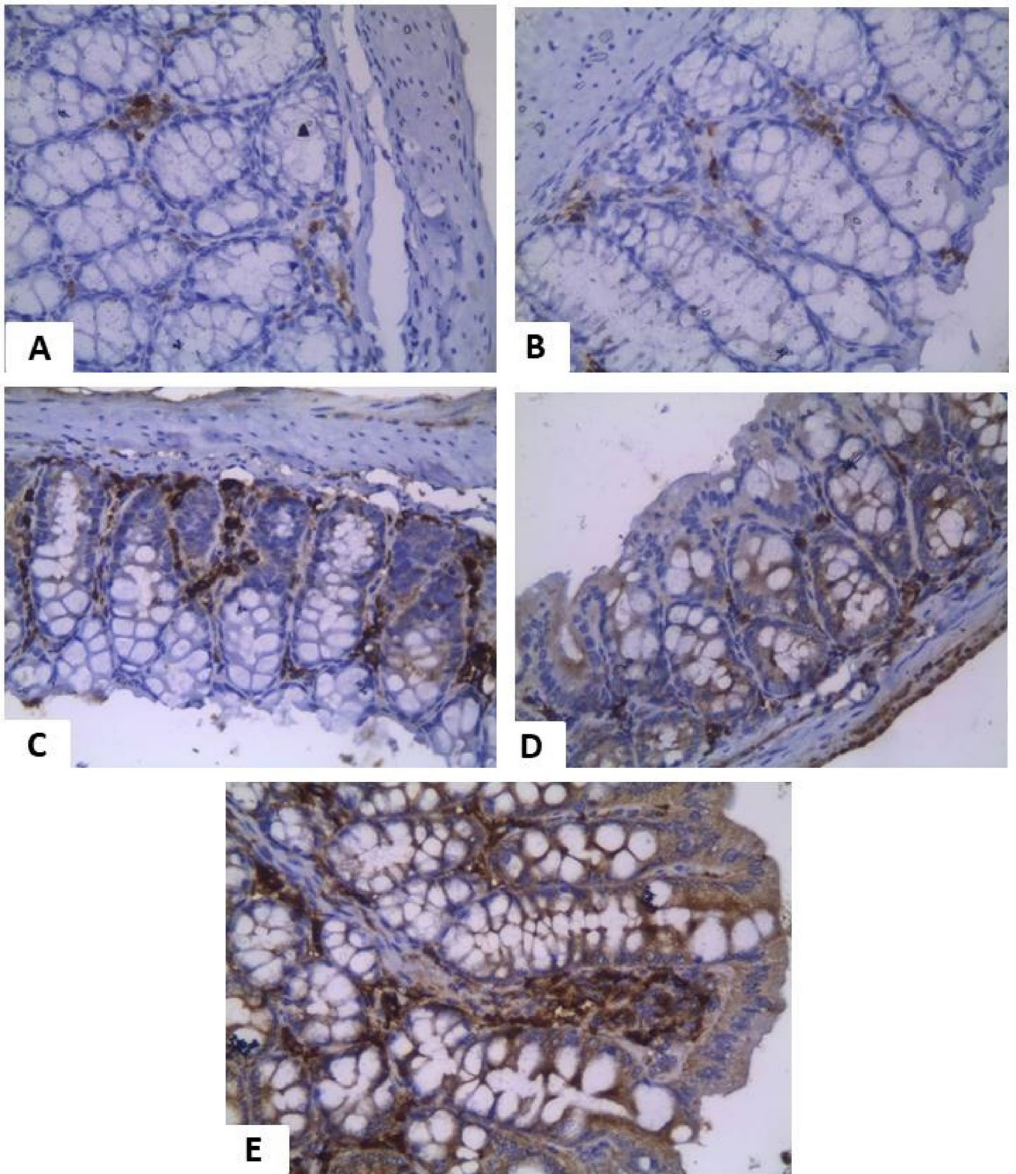
Immunohistochemical detection of IgA in the large intestine Groups **A** and **B** show no IgA-secreting cells. MTZ-treated tissue (**C**) shows mild focal IgA positivity. Probiotics-treated (**D**) reveals a mild to moderate increase in IgA cells. The combined treatment (**E**) shows a strong IgA response and evident inflammatory infiltration. (*All images at 400x magnification; H&E stain with nonspecific IgA staining in inflammatory cells.*)

As shown in Table 3; Figs. 6 and 7, Group I showed strong IgA expression, while Group II lacked IgA-secreting cells. MTZ (Group III) and probiotics (Group IV) partially restored IgA expression. The combined treatment (Group V) showed the highest IgA levels, similar to controls. Figure 6 (small intestine) and Fig. 7 (large intestine) confirm these patterns: Groups I and V showed strong staining, Groups III and IV showed mild to moderate expression, and Group II showed minimal or absent IgA

**Table 3:** Immunohistochemical findings of IgA secretory cells in the intestinal section

Group	Small intestinal expression	H Score	large intestinal expression	H Score
**Group I**	Negative	0	Negative	0
**Group II**	Negative	0	Negative	0
**Group III**	Mild positive	1+	Mild positive	1+
**Group IV**	Mild to moderate	1+	Mild to moderate	1+
**Group V**	Strong positive	2+	Strong positive	2+

### Serum cytokine levels

ELISA measurements of IL-1β, IL-6, and IFN-γ are summarized in Table 4.

- IL-1β: Elevated from 121.07 ± 13.73 (Group I) to 514.99 ± 32.18 (Group II). Reductions were observed in Groups III (29.1%), IV (49.7%), and V (66.9%) (*p* < 0.001).
- IL-6: Increased from 193.35 ± 18.55 (Group I) to 598.69 ± 45.50 (Group II), with subsequent reductions in Groups III (25.3%), IV (40.6%), and V (57.8%).
- IFN-γ: Rose from 155.56 ± 15.25 (Group I) to 779.09 ± 60.13 (Group II). Groups III, IV, and V showed reductions of 45.1%, 42.9%, and 60.1%, respectively. These results indicate that the combination therapy (Group V) exerted the strongest modulatory effect on the inflammatory cytokine response.

**Table 4:** The mean level of (IL-1B), (IL-6), and (IFN-γ) in serum of different groups

		Range	Mean ± SD	% of reduction	F. test	*p*. value	Post Hock test			
**IL-1**	Group I	103.59–134.81	121.07 ± 13.73		170.142	0.001*	P1	0.001*	P6	0.001*
	Group II	482.64–545.7	514.99 ± 32.18				P2	0.001*	P7	0.001*
	Group III	344.18–404.66	365.00 ± 27.23	29.1%			P3	0.001*	P8	0.001*
	Group IV	226.73–283.04	259.03 ± 26.59	49.7%			P4	0.011*	P9	0.001*
	Group V	153.37–190	170.69 ± 16.21	66.9%			P5	0.001*	P10	0.001*
**IL-6**	Group I	176.51–216.9	193.35 ± 18.55		93.416	0.001*	P1	0.001*	P6	0.001*
	Group II	542–653.33	598.69 ± 45.50				P2	0.001*	P7	0.001*
	Group III	404.68–500.92	447.33 ± 41.69	25.3%			P3	0.001*	P8	0.001*
	Group IV	317.18–384.8	355.51 ± 28.12	40.6%			P4	0.023*	P9	0.001*
	Group V	229.27–285.85	252.92 ± 24.31	57.8%			P5	0.001*	P10	0.001*
**IFN-γ**	Group I	134.85–170.03	155.56 ± 15.25		125.824	0.001*	P1	0.001*	P6	0.001*
	Group II	702.5–848.06	779.09 ± 60.13				P2	0.001*	P7	0.001*
	Group III	386.37–483.5	427.42 ± 47.28	45.1%			P3	0.001*	P8	0.552
	Group IV	389.11–470.45	445.08 ± 37.65	42.9%			P4	0.001*	P9	0.001*
	Group V	274.17–341.84	310.87 ± 29.99	60.1%			P5	0.001*	P10	0.001*

P1: G I & G II

P2: G I & G III

P3: G I & G IV

P4: G I & G V

P5: G II & G III

P6: G II & G IV

P7: G II & G V

P8: G III & G IV

P9: G III & G V

P10: G IV & G V

## Discussion

*Blastocystis* is a common intestinal protozoan with varied morphologies. The debate over its pathogenicity continues, with differences noted between asymptomatic and symptomatic isolates [3]. Metronidazole is the first-line treatment, but side effects, decreased effectiveness and resistance have been reported [31, 32]. Probiotics are gaining attention as alternative treatments for managing parasite replication and boosting immune responses, they have shown immunomodulatory effects against various pathogens like *Eimeria tenella*, trichinosis, *Toxoplasma gondii* and *Cryptosporidium parvum* [33–35].

To support the hypothesis that *L. fermentum and L. delbrueckii* exert their effect in situ through H₂O₂ production in the colon, it is important to demonstrate that *lactobacilli* are capable of colonizing this intestinal site. Previous human studies using colonic biopsies have shown that orally administered *lactobacilli*, such as *L. rhamnosus* GG, are able to attach to and transiently colonize the colonic mucosa [7]. Beyond colonization, accumulating evidence indicates that certain dairy-derived strains can exert strong anticolitic activity. In a mouse model of dextran sodium sulfate (DSS)-induced colitis, oral administration of *Lactobacillus delbrueckii* subsp. lactis CNRZ327 markedly ameliorated colonic inflammation, as evidenced by modulation of TGF-β, IL-6, and IL-12 levels in colonic tissue. These findings demonstrate that common dairy lactobacilli are not only capable of reaching the colonic environment but can also counteract inflammation and restore mucosal homeostasis, underscoring their therapeutic potential against colitis [8]. 

The observed increase in *Blastocystis* count and sustained viability in non-treated cultures over 48 h reflects the parasite’s natural growth under favourable conditions. This finding aligns with earlier studies [36, 37] which reported similar proliferation patterns, highlighting the organism’s capacity for survival and replication in vitro. MTZ demonstrated a marked inhibitory effect on *Blastocystis* growth, with reductions of 76.5% at 24 h and 88.6% at 48 h, alongside viability inhibition of 81.6% and 91.3%, respectively. These findings are in line with prior reports [37, 38], although some studies have noted emerging resistance [22, 39]. Lactobacillus probiotics also significantly reduced parasite growth (73.5% at 24 h and 79.4% at 48 h) with similar inhibition rates, supporting their antiparasitic potential [21, 40]. Notably, the combination of MTZ and probiotics produced the most substantial effect, reducing growth by 85.7% and 94.8% at 24 and 48 h, respectively, in line with studies advocating synergistic therapies [21].

Ultrastructural analysis using SEM showed variable surface morphologies in untreated *Blastocystis*, ranging from smooth to rough surface, consistent with previous reports [27]. MTZ treatment induced progressive damage, including membrane fragmentation, irregular ridges, and blebbing, especially after 48 h [41]. Lactobacillus-treated cultures exhibited milder alterations initially, with preserved spherical forms and limited fragmentation at 24 h, progressing to evident surface convolutions and membrane disruption by 48 h, aligning with earlier observations [42, 43]. When treated with both drugs, *Blastocystis* caused significant morphological alterations, including fragmentation and convolutions [44].

TEM analysis of untreated *Blastocystis* revealed normal morphology with distinct forms: the vacuolar form and granular form [45]. While MTZ treatment indicated programmed cell deaths such as cell shrinkage [46]. In cultures with *lactobacillus* probiotics, *Blastocystis* also showed apoptotic features aligning with [20].

When treated with both, *Blastocystis* displayed enhanced apoptotic features, suggesting improved efficacy against *Blastocystis* [20, 47]. This may be attributable to the mechanism of probiotics as spent media from probiotics can inhibit protozoan growth, with pH playing a significant role [48].

In the in vivo study, *Blastocystis* counts in the infected non-treated group averaged 148,540 ± 25,830.37/g stool, consistent with El-Sayed et al. (2017) and El-Askary et al. (2021) [22, 49]. MTZ-treated mice showed an 86.0% reduction in stool counts and 85.1% in intestinal content, aligning with El-Sayed et al. (2017), though some resistance was noted in another studies [21, 22, 50]. Probiotic-treated mice showed reductions of 84.1% in stool and 82.9% in intestinal content [2, 51]. Combined treatment achieved 98.5% reduction in both, confirming synergistic efficacy [20, 49].

Murine susceptibility to *Blastocystis* is strain dependent, with some strains (e.g., C57Bl/6) relatively refractory, whereas Swiss albino mice are permissive and develop intestinal pathology following inoculation [22]. This supports the appropriateness of our model for studying parasite–host interactions and therapeutic interventions.

Histopathology in the infected non-treated group showed mucosal ulceration, goblet cell increase, inflammation, villous loss, and brush border damage, consistent with previous reports [22, 52, 53]. MTZ-treated mice showed partial mucosal healing and moderate inflammation [54]. Probiotic treatment significantly restored villous structure and brush borders [20, 54, 55]. Combined therapy led to nearly complete healing with normal villi and no inflammation, confirming efficacy [20, 55]. These findings support the beneficial role of probiotics against *Blastocystis*. Audebert et al. (2016) highlighted the importance of stable gut microbiota for intestinal health, suggesting probiotic-rich diets may help resist protozoan colonization [2]. Dinleyici et al. (2011) also reported that probiotics exert immunomodulatory effects and strengthen the mucosal barrier, enhancing gut defence [56]. 

Immunohistochemical analysis revealed low secretory IgA levels in both non-infected and infected non-treated groups [22]. MTZ and Lactobacillus-treated groups showed mild to moderate IgA expression, consistent with previous findings [22, 55]. The combined treatment group exhibited high IgA levels, suggesting enhanced mucosal immunity [45, 55]. Biochemically, the infected non-treated group showed significant increases in IL-1β, IL-6, and IFN-γ compared to controls [19, 30]. MTZ treatment reduced these cytokines by 29.1%, 25.3%, and 45.1%, respectively, consisting with previous reports [57, 58]. Lactobacillus probiotics reduced IL-1β, IL-6, and IFN-γ by 49.7%, 40.6%, and 42.9%, respectively [59, 60]. The combined MTZ and probiotic group showed the most marked reductions (66.9%, 57.8%, 60.1%) [61, 62]. Prior studies also noted probiotic benefits in IBD-related *Blastocystis* infections, with increased IL-10 and TGF-β levels [60, 63].

## Conclusion

Probiotics, demonstrate promising therapeutic potential against *Blastocystis* by modulating immune responses, reducing inflammation, and enhancing gut barrier integrity. Incorporating these strains into dietary regimens or supplements may offer an effective adjunct or alternative to conventional treatments, contributing to improved gut health and pathogen control.

## Data Availability

The datasets used and/or analysed during the current study are available from the corresponding author on reasonable request.

## Declarations

### Study limitation

SEM images are representative and provide qualitative evidence only. The proportion of cysts showing each morphological change was not quantified, limiting the ability to generalize the findings. Additionally, molecular subtyping of *Blastocystis* was not performed, which restricts conclusions regarding strain-specific pathogenicity. Future studies should include systematic quantification of morphological alterations and parasite subtyping to strengthen the interpretation and relevance of the results.
